# High risk of asthma among early teens is associated with quantitative differences in mite and cat allergen specific IgE and IgG4: a modified Th2 related antibody response revisited

**DOI:** 10.1016/j.ebiom.2024.105556

**Published:** 2025-02-01

**Authors:** Thomas A. Platts-Mills, Behnam Keshavarz, Jeffrey M. Wilson, Sheryl L. Rifas-Shiman, Samuel M. Ailsworth, Joanne E. Sordillo, Lisa Workman, Martin Chapman, Jonas Lidholm, Emily Oken, Diane R. Gold

**Affiliations:** a Division of Allergy & Clinical Immunology, Department of Medicine, University of Virginia, Charlottesville, VA, USA; b The Department of Population Medicine, Harvard Medical School and Harvard Pilgrim Health Care Institute, Boston, MA, USA; c InBio, Charlottesville, VA, USA; d Thermo Fisher Scientific, Uppsala, Sweden; e The Channing Division of Network Medicine, Department of Medicine, Brigham and Women's Hospital and Harvard Medical School, Boston, MA, USA; f The Department of Environmental Medicine, Harvard T.H. Chan School of Public Health, Boston, MA, USA

**Keywords:** Component allergens from cat and mite, Asthma, Early teens, IgE and IgG4 antibodies, Less abundant allergens in house dust

## Abstract

**Background:**

Although proteins derived from cats are an important contributor to indoor allergen exposure in relation to asthma, it has been known for at least twenty years that some children who live in a house with a cat can become clinically tolerant to these animals. In 2001, we reported that children exposed to high levels of cat allergens made high levels of IgG4 antibodies to the cat allergen Fel d 1, and we coined the term “a modified Th2 response”. However, this phenomenon is still poorly understood.

**Methods:**

We studied serum antibodies among 616 individuals in the Viva unselected birth cohort recruited at their early teen visit (mean age 13.1 SD 0.8). IgE and IgG4 antibodies were measured by ImmunoCAP to inhaled allergens as well as the best characterised component allergens of cat, Fel d 1, Fel d 2, Fel d 4, and Fel d 7, and the dust mite allergens Der p 1, Der p 2, Der p 10, and Der p 23.

**Findings:**

The results confirm that young teens living in a home with a cat make high levels of IgG4 specific for cat allergens, and that those antibodies, and specifically those to Fel d 1 are negatively associated with asthma. By contrast, the IgG4 responses to Fel d 4 and Fel d 7 are significantly lower and have no significant association with asthma. Perhaps more surprisingly, a similar effect is seen in relation to dust-mite allergens. Although the allergen Der p 1 is a major part of the IgE response to mite allergens, this protein also induced high prevalence and levels of IgG4 antibodies and has a less strong relationship to asthma than IgE to Der p 2 or Der p 23. Indeed, values of specific IgE to Der p 1 >3.5 IU/mL were not significantly related to asthma (OR 1.5 CI 0.8–2.8, p = 0.3, Chi^2^ test). The prevalence and levels of specific IgG4 to these less abundant allergens are significantly lower for Der p 2 and almost absent for Der p 23.

**Interpretation:**

High exposure to specific allergens in household dust can enhance production of both sIgE and sIgG4 antibodies, while allergens where abundance is significantly lower in dust can induce sIgE with limited or no sIgG4. The result is that the less abundant allergens, i.e., Fel d 4, Fel d 7, Der p 2, and Der p 23, may have a significantly higher relevance to asthma than expected because they induce less sIgG4.

**Funding:**

This work was funded by R01-AI20565 (TPM) and support for the IgE and IgG4 assays provided by 10.13039/100011033Phadia/Thermo Fisher Kalamazoo, Michigan. Project Viva is also supported by NIH R01HD034568 and R24ES.

**Research in context**

**Evidence before this study**

Evidence has come from studies in Sweden, New Zealand, and the USA, that many children who live in a home with a cat become clinically tolerant to allergens derived from cats. This could occur, despite having made both sIgE and sIgG4 to the major allergen of cat (Fel d 1). However, the details of the response induced by exposure to high quantities of cat allergen were not well understood. In particular, the relationship between asthma and the immune response to component allergens of cat and dust mites had not been defined. In part previous studies were limited because the assays used for sIgG4 were only semi-quantitative, and there was limited information about exposure to allergens other than Fel d 1 from cat and Der p 1, from dust mites.

**Added value of this study**

The primary value of the present study comes from two findings. Firstly, confirmation that the quantities of sIgG4 to the major allergens of both cat and dust mite are relevant to understanding the relationship of these allergens to asthma. For both cat extract and Fel d 1 a high ratio of sIgG4 to sIgE is associated with a significantly decreased (p = 0.009, Mann Whitney U test) association with asthma. Equally, the results show that the reduced association between sIgE to Der p 1 and asthma correlates with a high prevalence of sIgG4 to Der p 1. Secondly, for the more recently identified and less abundant allergens from both cat and mite there is less sIgG4 compared to sIgE. In keeping with that sIgE to those allergens is significantly positively associated with asthma.

**Implications of all the available evidence**

For the “minor” allergens quantitative exposure is lower than that for those allergens which were identified first, but their association with asthma is stronger. In turn the current evidence argues that component spreading for sIgE antibody responses to cat and mite allergens can occur with levels of exposure to the relevant proteins that are lower than those necessary to induce sIgG4 to these allergens.

## Introduction

The relevance of both indoor and outdoor allergens to asthma among children and teenagers has been known from as early as the 1970's.^1^, ^2^, ^3^ Furthermore, in most studies, higher overall exposure to allergens in a community or in individual homes has been found to correlate with increased prevalence of sensitisation.^4^^,^^5^ Thus, it was a surprise when Hesselmar and his colleagues in Sweden presented data showing that children living in a house with a cat were less likely to become sensitised to, or symptomatic on exposure to, cat allergens.^6^ However, that publication led other authors in the field to evaluate their own results, which in some but not all cases led to similar findings for the effect of cat ownership.^7^ The possible negative effects of high exposure to cat allergens can only be recognised if many participants in the same community who do not live in a home with a cat become sensitised. This sensitisation in turn depends on the characteristics of cat allergens which can lead to their presence in schools or homes without a cat.^8^^,^^9^ The presence of allergens outside homes with a cat is well recognised but this is strongly influenced by the prevalence of cats in the homes within a community, which ranges from 5% in Spain to ≥50% in New Zealand.^10^^,^^11^ In our study on middle school children in the USA, we found both a lower prevalence of specific IgE (sIgE) to cat and an increased prevalence of specific IgG4 (sIgG4) antibodies to the cat allergen Fel d 1 in the sera of children who lived with high exposure to cat allergen and did not have asthma. At that time, the measurements of sIgG and sIgG4 were made with an antigen binding radioimmunoassay which was technically difficult. Consequently, it was not possible to compare units for sIgG4 and sIgE accurately.^7^ However, in 2006 a similar phenomenon of high sIgG4 and decreased sIgE was reported among animal house technicians who tolerated working with rats and mice despite high exposure to the relevant allergens.^12^

The IgG4 isotype is well recognised as an element of Th2 immune responses, and is considered to play a role in the response to allergen-specific immunotherapy and other forms of tolerance.^13^ Like IgE, IgG4 class switch depends on IL-4, in addition, production of IgG4 is increased in situations where there is concomitant IL-10 production.^14^, ^15^, ^16^, ^17^ The relevance of cat ownership to the effect of IgG4 has been studied in detail by Dr. Wambre and his colleagues on a small number of subjects in Seattle.^16^ Antibodies of the IgG4 isotype do not activate complement, only bind to Fc receptors with low affinity and can rearrange their antigen binding by so-called Fab-arm exchange. These factors are all considered to contribute to their anti-inflammatory effects.^18^ Interestingly, sIgG4 responses appear to require higher levels of exposure and to decline rapidly if there is a reduction in exposure to the relevant allergens.^19^ This difference may reflect the fact that IgG4 responses do not include long lived plasma cells in the bone marrow which are a feature of sIgE responses. Given that IgG4 contributes to allergen tolerance, the prevalence and quantities of specific IgG4 (sIgG4) to both allergen extracts and components may influence the relationship between sIgE to these proteins and asthma. Equally, the quantities of the specific allergen proteins derived from sources in the indoor environment may be relevant to the production of sIgE and sIgG4.

The first major allergens of cat (Fel d 1) and dust mite (Der p 1) were purified in the 1970's and led to measurements of these allergens in house dust and airborne.^20^, ^21^, ^22^, ^23^ More recently, multiple allergens have been identified, cloned and expressed as recombinant allergens. However, for most of the allergens identified more recently there is little, or no data published about exposure in homes. Where measurements have been made, it appears that the allergens identified more recently are present in smaller quantities.^22^^,^^24^ In the present study we evaluated sIgE antibodies to a range of inhalant allergens in sera from 616 participants in the Viva birth cohort obtained at the time of their “early teen” visit (mean age 13.1 years).^25^^,^^26^ Given that cat and mite sensitisation were consistently associated with asthma, we used ImmunoCAP assays to measure sIgE and sIgG4 to both allergen extracts and relevant component allergens from both of these sources. Those assays use the same solid phase for binding antibodies of the two isotypes and the results can be expressed in absolute units such as μg/mL or ng/mL.^27^^,^^28^ Our objective was to further understand the relationship between sIgE to mite and cat allergens and asthma. In particular, to carry out quantitative assays of sIgE and sIgG4 to the components from these sources, focussing on those allergens where there is information about quantitative exposure in homes.

## Methods

### Study participants

**Fig. 1:**
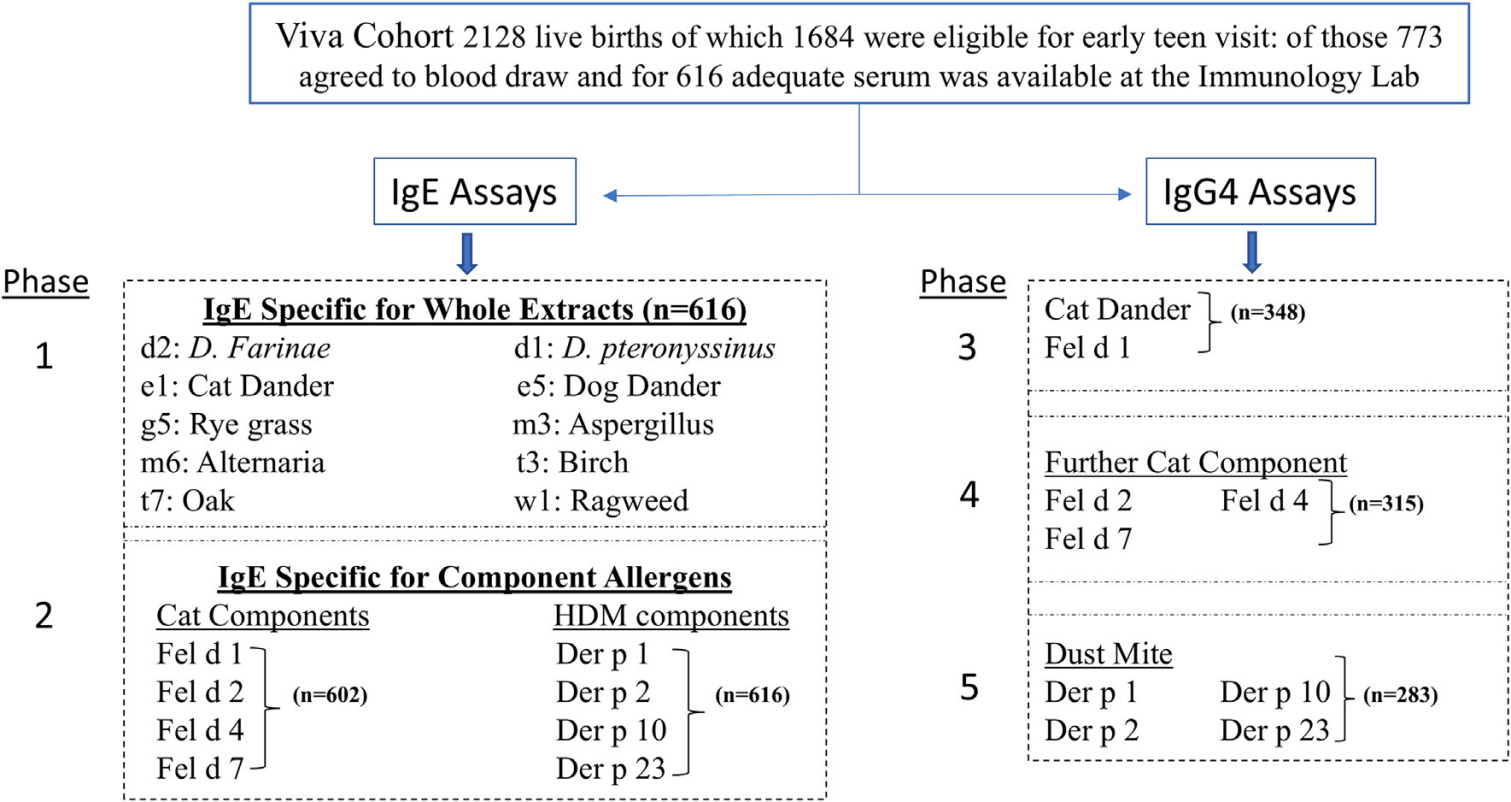
**Flow diagram for assays for IgE and IgG4**. The samples from 616 participants in the project viva birth cohort obtained during their early teen visit, came in the form of heparinised plasma. Work in our laboratory on ImmunoCAP antibody assays over the last few years has established that this form of plasma gives results for both IgG4 and IgE antibodies that are ≤5% different from results using serum. The samples are referred to in the text as sera because the study of these assays is called serology.

We analysed samples from 616 children who were enrolled in Project Viva. This is an ongoing longitudinal pre-birth cohort originally recruited in eastern Massachusetts in which no selection criteria in relation to asthma or allergic disease were used at the time of enrollment.^25^ Of the 2128 live born infants in the cohort, we had information on asthma status of 1130, and 773 participants attended for early teen visit and blood draw. Of these 616 had adequate serum for measurements of total IgE, and sIgE to extract allergens. The design of this cross-sectional study and the details of the assays are shown in Fig. 1. Both the questionnaire data on asthma and the serum samples were obtained at the time of the early teen visit.

### Ethics

At each visit, we obtained written informed consent from the mothers, and beginning in mid-childhood verbal assent from the child through age 18 years, after which we obtain written informed consent from offspring themselves. The Harvard Pilgrim Health Care Institutional Review Board approved all study protocols in line with ethical standards established by the Declaration of Helsinki. Project Viva also has a record on ClinicalTrial.gov (record ID NCT02820402). The studies on de-identified sera in Virginia had the IRB approval number of IRB-17026. The project Viva data repository number was IRB-Nett228471.

### Assessment of asthma and asthma severity

In this study, we defined current asthma (asthma) (n = 86) as present if a mother reported at the early-teen visit that the participant had physician-diagnosed asthma, and in addition reported either use of asthma medication or wheezing in the past 12 months.^25^^,^^26^ Additionally, we defined moderate to severe asthma (MSA; n = 45) as participants with asthma whose parents reported two or more acute episodes of asthma in the past 12 months. The no asthma comparison group included never asthma, but also included ever asthma that was not current.

### Measurement of IgE and IgG4 to specific allergens (see Fig. 1)

All sIgE and sIgG4 assays were completed using the ImmunoCAP 250 instrument and commercially available ImmunoCAPs (Thermo Fisher Scientific/Phadia, Kalamazoo, MI). The catalogue number for the IgE specific conjugate is 10-9310-02. As part of our plan to conserve samples, the sera were assayed for total serum IgE; the Phadiatop screening assay for inhalant allergens; and the fx5 assay for IgE to food allergy. These three screening assays were carried out on undiluted samples. (i) Total serum IgE. (ii) Phadiatop which is a proprietary ImmunoCap to screen for IgE to inhalant allergens (regionally relevant pollens, moulds, and indoor allergens including mite and animal dander). The results are reported as response units and scored positive or negative. (iii) The ImmunoCAP fx5 carries a mix of food allergens including egg white, peanut, wheat, cow's milk, fish, and soy, and is scored positive or negative. Each of the samples were graded positive if either of the two allergen mix ImmunoCAPs was positive or the total IgE was ≥120 IU/mL. On this basis we identified 176 sera for which all three screening tests were negative.

The sera with positive results from the screen were assayed for sIgE to extracts of the allergens: *Dermatophagoides farinae*, *Dermatophagoides pteronyssinus,* cat dander, dog dander, Aspergillus, Alternaria, as well as pollens of rye grass, birch, oak, and common ragweed. Remaining serum from nearly all (n = 616) participants, including all 86 asthmatics, was available to assay sIgE to cat components (Fel d 1, Fel d 2, Fel d 4, and Fel d 7), as well as *D. pteronyssinus* components: (Der p 1, Der p 2, Der p 10, and Der p 23).^29^, ^30^, ^31^, ^32^, ^33^ IgE to foods were assessed by measuring IgE to the Phadia fx5 cap, which contains a mixture of food allergens, and sera were classified as positive or negative by the ImmunoCAP instrument. Specific IgE assays to the component allergens from cat and dust mite were carried out on sera from all participants with asthma (n = 86) and on sera from controls without asthma who tested positive, ≥0.35 IU/mL for sIgE to cat or dust mite allergens. The values for sIgE to component allergens for both the cat and the mite were assumed to be negative if the relevant extracts assays were negative.

#### Specific IgG4 assays

The specific IgG4 (sIgG4) assays for cat and mite extracts as well as for the relevant components used the same ImmmunoCAPs as the sIgE assays. The IgG4 assays used isotype specific reagents provided by Phadia; the IgG4 conjugate catalogue number is 10-9549-02. In the first phase of the IgG4 assays we tested sIgG4 to cat dander extract and to the major cat allergen, Fel d 1. Those assays were carried out on all participants with asthma where data on cat ownership was available, and also on 264 participants without asthma including 114 with a cat at home. Due to limited funds and insufficient remaining sample volume for some participants, we restricted testing of specific IgG4 to cat and mite component allergens to two groups: (i) all participants with asthma (“cases”) and (ii) a random sample of participants without asthma (“controls”). We randomly selected two controls for each case. Cases and controls were not matched on any characteristics.

#### Specific IgG4 to *D. pteronyssinus* and mite components

The assays were carried out on mite extracts as well as the component mite allergens Der p 1, Der p 2, Der p 10, and Der p 23. These were carried out on the available sera from participants with asthma and on 283 controls which included 141 sensitised and 142 not sensitised to mite. The non-sensitised group included 35 of the participants who had tested negative in the pre-screening for IgE antibodies.

The decisions about which components of mite and cat to study were based on three factors: firstly, they were available as recombinant allergens on ImmunoCAP; secondly they were well recognised and included major allergens associated with asthma^34^^,^^35^; and thirdly, for Der p 1, Der p 2, and Der p 23 as well as Fel d 1 and Fel d 4, there are published estimates of the quantities of these allergens in homes.^24^^,^^33^ For cat and mite allergen components, sIgE was considered negative if sIgE to the corresponding allergen extract was below the limit of detection (LOD). We did not assume that any sIgG4 assays were negative unless they had been measured. The LOD used for sIgE and sIgG4 antibodies were 0.35 IU/mL and 0.07 μg/mL, respectively. For comparisons between sIgE and sIgG4, results were converted to ng/mL for both isotypes (for sIgE one IU/mL = 2.4 ng/mL, for sIgG4 one μg/mL = 1000 ng/mL).

### Limits of detection for antibody assays (LOD)

The LOD values of the assays for sIgE and sIgG4 are based on values recommended by Thermo/Fisher/Phadia during the time that we have been studying Project Viva. In addition, we adjusted LOD values because of dilution of the sample for sIgE assays and our own interpretation of the significance. The LOD for IgE antibody assays carried out with ImmunoCAP was initially 0.35 IU/mL, but in 2012 the company adopted a lower value of 0.1 IU/mL. As outlined above, the sera were diluted 1:2 before carrying out sIgE assays and in theory we could have adopted an LOD of 0.2 IU/mL after correction for the dilution factor, but chose to use 0.35 which we have used extensively in the past. Given that the International Unit (IU) for IgE ∼ 2.4 ng, 0.35 IU/mL equals 0.84 ng/mL.

Our own calculations of appropriate values for sIgG4 to allergens have consistently been below 0.1 μg/mL and we adopted a value of 0.07 μg/mL or 70 ng/mL. In the past the company has recommended an LOD value of 0.3 μg/mL (300 ng/mL). We have continued to use the LOD value of 70 ng/mL. Although we have consistently used the LOD of 70 ng/mL in those figures that include data on sIgG4 in ng/mL. It is important to note that in data for patients with eosinophilic esophagitis (EoE), it is not unusual to find sIgG4 values for milk or wheat components ≥100 μg/mL (Fig S2).

### Statistics

Antibody levels were expressed as geometric mean (GM) of the positive values with 95% confidence intervals (95% CI), and compared using Mann–Whitney U test. Other continuous variables were compared using Student's t test, as appropriate. Chi-squared test was used for bi-variate comparison of categorical variables. Results were expressed as odds ratios (OR) with 95% CI. The odds ratios were adjusted for child age, sex, body mass index (BMI), delivery by C-section, household income, smoker at home, cat at home, dog at home. (Adjusted without including race or ethnicity). All analyses were performed using Stata V.15.0 (StataCorp LP, College Station, TX) and GraphPad Prism V.9.3 (GraphPad Software, La Jolla, CA). Assays for both sIgE and sIgG4 to some of the allergens reported have had a prevalence of positives as low as 5% and as high as 90%. This creates questions about the analysis of levels of the positives. In general, we allotted values to the negative results of half the LOD values if the prevalence of negatives was ≤20% of the total samples. In situations where there was a higher prevalence of negatives, we examined prevalence using Chi square analysis and evaluated the levels as the GM and 95% confidence intervals of the positives.

## Results

### Characteristics of the cohort

Characteristics of participants in the present study are summarised in Table 1. Mean age of the cohort was 13.1 ± 0.8 years (range 12–16). Eighty-six participants (14%) had asthma with male and female children equally represented in this group. The proportion of both Black participants and those of “Other races” with asthma compared to White participants with asthma was significantly higher (p = 0.02) (see Table 1). Children with asthma and without asthma were similar with respect to age, sex, household income (>$70,000), birth delivery (%C-section), body mass index (BMI), living with a smoker, and living with a pet(s) (cat and/or dog) at home. Among the 86 with asthma, 74% were sensitised to at least one of the inhalant allergens. Total IgE concentrations were also significantly higher in the group with asthma (GM 110 IU/mL vs 55 IU/mL) (p < 0.001, Student T test). Of the 86 children with asthma, 45 had moderate/severe asthma (MSA) on the basis of reporting two or more attacks of asthma in the previous 12 months.^26^ Total IgE (GM 162 IU/mL vs 55 IU/mL) and sIgE prevalence to inhalant (82% vs 53%) or food (60% vs 27%) allergens were also significantly higher in the MSA group compared to the group with no asthma (p < 0.001, Chi^2^ test) (Table 1).

**Table 1:** Characteristics of participants in the cohort enrolled at the early-teen visit in Project Viva.

Characteristic	Overall n = 616	No asthma (n = 530)	Asthma (n = 86)^a^	p value^b^	MSA^c^ (n = 45)	p value^d^
Demographic characteristics						
Age, median years (range)	12.8 (11.9–16.4)	12.9 (11.9–16.4)	12.8 (12.1–15.0)	0.35^g^	12.7 (12.1–15.0)	0.11^g^
Sex: male, n (%)	322 (52.3)	279/530 (52.6)	43/86 (50.0)		22/45 (48.9)	
Sex: female n (%)	294 (47.7)	251 (47.4)	43 (50.0)	0.73^f^	23/45 (51.1)	0.63^f^
Race/ethnicity						
White, n (%)	381 (61.9)	339 (64.0)	42 (48.8)	Ref	23 (51.1)	Ref
Black, n (%)	107 (17.4)	86 (16.2)	21 (24.4)	0.02^f^	9 (20.0)	0.37^f^
Hispanic, n (%)	26 (4.2)	23 (4.3)	3 (3.5)	>0.99^f^	2 (4.4)	0.69^f^
Others,^e^ n (%)	102 (16.6)	80 (15.1)	20 (23.3)	0.03^f^	11 (24.4)	0.99^f^
House income (>$70K)	355/552 (64.3)	308/476 (64.0)	47/76 (61.8)	0.56^f^	26/40 (65.0)	>0.99^f^
C-section, n (%)	138/616 (22.4)	113/530 (21.3)	25/86 (29.1)	0.12^f^	12/45 (26.7)	0.45^f^
BMI (kg/m^2^), mean (SD)	21.0 (4.5)	20.8 (4.2)	22.4 (5.9)	0.07^g^	21.8 (6.8)	0.62^g^

Characteristic	Overall n = 616	No asthma (n = 530)	Asthma (n = 86)	p value^b^	MSA^c^ (n = 45)	p value^d^
Current factors related to asthma risk						
Smoker in home, n (%)	69/613 (11.3)	60/527 (11.4)	9/86 (10.5)	>0.99^f^	5/45 (11.1)	>0.99^f^
Cat at home, n (%)	179/599 (29.9)	148/516 (28.7)	31/83 (37.3)	0.12^f^	17/45 (37.8)	0.23^f^
Dog at home, n (%)	234/606 (38.6)	201/522 (38.5)	33/84 (39.3)	0.90^f^	21/44 (47.7)	0.26^f^
Total IgE (IU/mL)	60.5 (53.6–68.3)	55.0 (48.5–62.3)	110 (75.5–159)	<0.001^g^	162 (98.1–266)	<0.001^g^
sIgE to any inhalant,^h^ n (%)	347/616 (56.3)	283/530 (53.4)	64/86 (74.4)	<0.001^f^	37/45 (82.2)	<0.001^f^
sIgE to any food,^i^ n (%)	187/616 (30.4)	144/530 (27.2)	43/86 (50)	<0.001^f^	27/45 (60.0)	<0.001^f^

a Current asthma (asthma) defined based on early-teen visit and maternal report of ever diagnosed with asthma by healthcare professional plus taking asthma medication in the past 12 months or wheezing symptoms in 12 months prior to early-teen visit.

b Analysis is between asthma vs no asthma groups.

c Moderately Severe Asthma (MSA) defined as children with asthma who have had ≥2 episodes of asthma attacks in the past 12 months.

d Analysis is between MSA vs no asthma groups.

e Others: includes Asians, children with more than 1 race or other.

f Fisher's exact test.

g Mann–Whitney U test was used to compare the means.

h Inhalant allergens include house dust mite, cat, dog, rye grass, Aspergillus species, Alternaria species, birch, oak, and ragweed.

i Screening was performed using the Phadia Fx5 food mixture cap.

### Sensitisation to inhalant allergens and risk of asthma

**Fig. 2:**
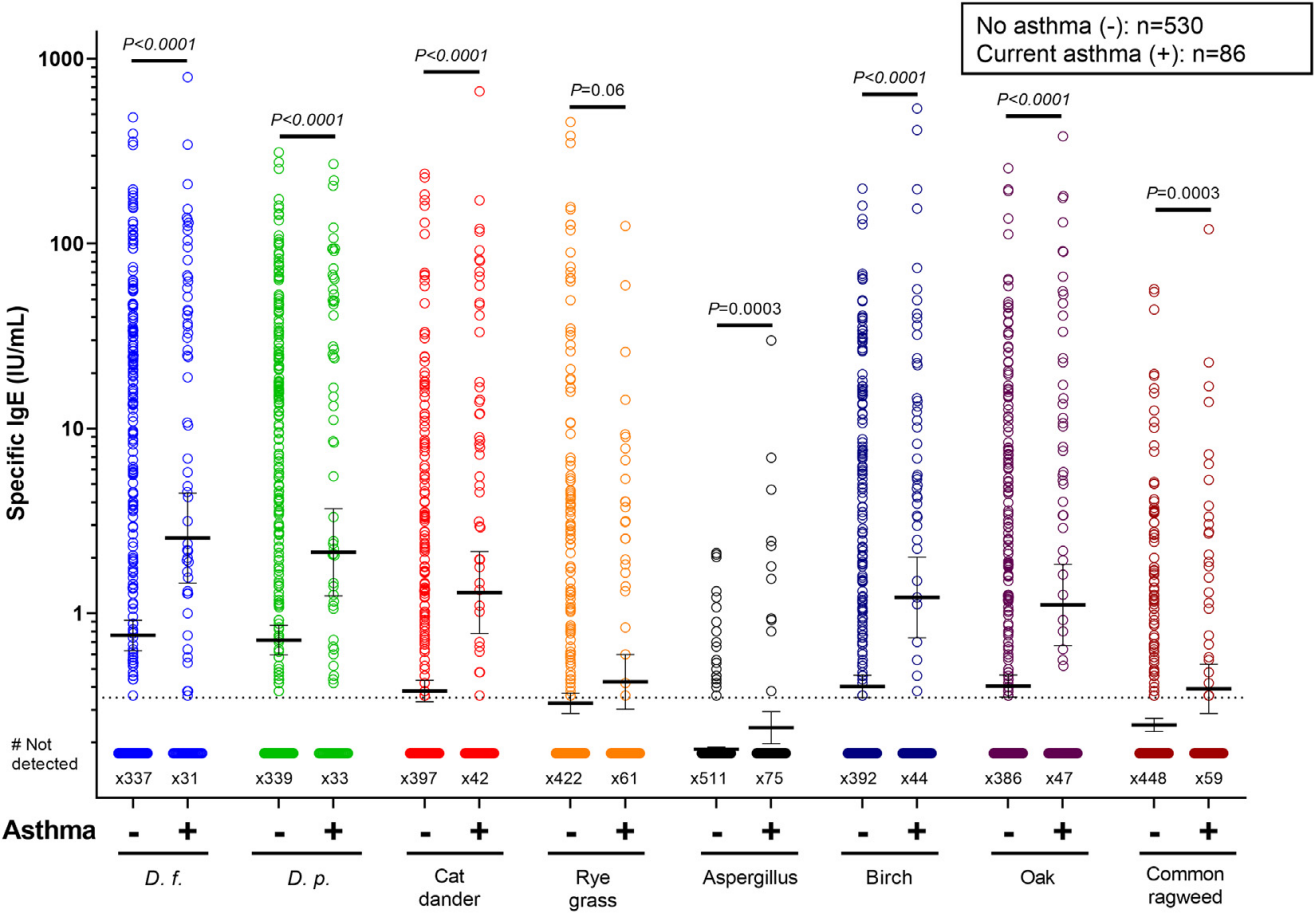
Levels of sIgE to 8 inhalant allergens. Numbers below the dotted line indicate values below LOD (<0.35 IU/mL). Values expressed as geometric mean (95% CI) and compared by the Mann–Whitney U test.

The prevalence of sensitisation to the 10 inhalant allergens tested was high in the cohort. Participants with asthma, compared to no asthma, had significantly higher levels of sIgE to mite, cat dander, dog dander, and aspergillus, and also to the pollens of silver birch, oak and common ragweed (Fig. 2, Table 2). The positive association between mite sensitisation and asthma was less strong than in some previous studies.^36^ In particular, the number of participants without asthma who had sIgE to mite ≥0.35; ≥3.5 or ≥50 IU/mL was 191 (36%); 130 (24.5%); and 33 (6.2%) respectively. Nonetheless, the highest levels of sIgE (i.e., ≥50 IU/mL) to mite (*D. farinae*, or *D. pteronyssinus*) and cat dander, as well as oak and birch pollens were strongly associated with both asthma and MSA (See bold values in Table 2).

**Table 2:** Relationship of sIgE levels in early teens with current asthma (asthma), moderate to severe asthma (MSA), and no asthma.

Specific IgE	Allergens	No asthma	Asthma	Prevalence		Prevalence		
				Adjusted OR^d^	p value^a^	MSA	Adjusted OR^d^	p value^b^
		(n = 530)	(n = 86)			(n = 45)		
>0.35 IU/mL	Dust mite (d1)^c^	191 (36%)	53 (61.6%)	3.1 (1.8, 5.3)	<0.001	30 (66.7%)	3.5 (1.7, 7.0)	<0.001
	Cat dander (e1)	133 (25.1%)	44 (51.2%)	3.6 (2.1, 6.2)	<0.001	29 (64.4%)	6.1 (3.0, 12.7)	<0.001
	Dog dander (e5)	117 (22.1%)	45 (52.3%)	4.8 (2.7, 8.3)	<0.001	29 (64.4%)	7.5 (3.6, 15.5)	<0.001
	Rye grass (g5)	108 (20.4%)	25 (29.1%)	1.6 (0.9, 2.9)	0.07	13 (28.9%)	1.8 (0.9, 3.9)	0.18
	Aspergillus (m3)	19 (3.6%)	11 (12.8%)	5.9 (2.5, 14.2)	<0.001	8 (17.8%)	8.0 (3.0, 21.4)	<0.001
	Alternaria (m6)	33 (6.2%)	10 (11.6%)	2.8 (1.2, 6.3)	0.05	7 (15.6%)	3.5 (1.4, 9.1)	0.03
	Silver birch (t3)	138 (26%)	42 (48.8%)	2.8 (1.6, 4.7)	<0.001	24 (53.3%)	3.8 (1.9, 7.6)	<0.001
	Oak (t7)	144 (27.2%)	39 (45.3%)	2.1 (1.2, 3.6)	<0.001	22 (48.9%)	2.5 (1.3, 5.0)	0.003
	Ragweed (w1)	82 (15.5%)	27 (31.4%)	2.6 (1.4, 4.7)	<0.001	12 (26.7%)	2.1 (0.9, 4.6)	0.06
>3.5 IU/mL	Dust mite (d1)^c^	130 (24.5%)	32 (37.2%)	2.1 (1.2, 3.7)	0.01	21 (46.7%)	3.2 (1.6, 6.3)	0.002
	Cat dander (e1)	59 (11.1%)	28 (32.6%)	4.3 (2.3, 8.1)	<0.001	20 (44.4%)	7.5 (3.5, 15.9)	<0.001
	Dog dander (e5)	29 (5.5%)	20 (23.3%)	5.3 (2.5, 11.2)	<0.001	14 (31.1%)	8.9 (3.8, 20.9)	<0.001
	Rye grass (g5)	48 (9.1%)	12 (14%)	2.1 (1.0, 4.4)	0.16	7 (15.6%)	2.5 (1.0, 6.4)	0.18
	Aspergillus (m3)	0 (0%)	3 (3.5%)	–	–	3 (6.67%)	–	–
	Alternaria (m6)	14 (2.6%)	5 (5.8%)	3.2 (1.0, 9.9)	0.12	4 (8.9%)	5.0 (1.4, 17.7)	0.04
	Silver birch (t3)	70 (13.2%)	30 (34.9%)	3.8 (2.1, 6.8)	<0.001	18 (40.0%)	5.3 (2.6, 10.8)	<0.001
	Oak (t7)	70 (13.2%)	27 (31.4%)	3.7 (2.0, 6.6)	<0.001	16 (35.6%)	4.6 (2.2, 9.7)	<0.001
	Ragweed (w1)	22 (4.2%)	8 (9.3%)	2.8 (1.0, 7.6)	0.05	6 (13.3%)	4.2 (1.4, 12.8)	0.02
>50 IU/mL	Dust mite (d1)^c^	33 (6.2%)	15 (17.4)	**4.5 (2.1, 9.9)**	**<0.001**	10 (22.2%)	**7.3 (2.9, 18.3)**	**<0.001**
	Cat dander (e1)	12 (2.3%)	10 (11.6%)	**7.7 (2.7, 21.6)**	**<0.001**	8 (17.8%)	**10.5 (3.5, 32.1)**	**<0.001**
	Dog dander (e5)	1 (0.2%)	2 (2.3%)	–	–	1 (2.2%)	–	–
	Rye grass (g5)	14 (2.6%)	2 (2.3%)	1.2 (0.3, 6.1)	>0.86	2 (4.4%)	2.6 (0.5, 13.1)	0.4
	Aspergillus (m3)	0 (0%)	0 (0%)	–	–	0 (0)	–	–
	Alternaria (m6)	2 (0.4%)	0 (0%)	–	–	0 (0)	–	–
	Silver birch (t3)	8 (1.5%)	6 (7%)	**5.0 (1.5, 16.1)**	**<0.001**	5 (11.1%)	**8.8 (2.4, 32.5)**	**0.002**
	Oak (t7)	8 (1.5%)	9 (10.5%)	**9.1 (3.1, 27.0)**	**<0.001**	7 (15.6%)	**15.1 (4.4, 51.3)**	**<0.001**
	Ragweed (w1)	2 (0.4%)	1 (1.2%)	–	–	1 (2.2%)	–	–

Bold values show adjusted odds ratios, confidence intervals, and p values (Chi^2^ test) for positive relationships to asthma and MSA identified in the text.

a Analysis is between no asthma vs asthma groups.

b Analysis is between MSA vs no asthma groups.

c *D. pteronyssinus*.

d Adjusted for child age at the Early Teen Visit, sex, race and ethnicity, BMI, delivery by cesarean section, household income, smoker in the home, cat at home, and dog at home.

### Specific IgE (sIgE) to component allergens from cat and dust mite

Because mite and cat dander were the two major sources of indoor allergens and both were significantly associated with asthma, we further analysed sera from participants with and without asthma for sIgE to cat and mite components (See Table 2). The component assays using the ImmunoCAP were available for four well-established allergens from cat, Fel d 1, 2, 4, and 7, as well as Der p 1, 2, 10, and 23 from mite.^34^ The prevalence of sIgE to cat components among participants with asthma was highest for Fel d 1 (50%) followed by Fel d 4 (25.6%), Fel d 7 (19.8%), and Fel d 2 (10.5%) and these values were significantly higher than those for the group with no asthma (Table 3). In particular, the association between asthma and sIgE to Fel d 1 and Fel d 4 remained strong for levels >50 IU/mL (OR 6.0, p < 0.001 and OR 11.3, p = 0.01 respectively, Chi^2^ test). For the mite allergen components, the highest prevalence of sIgE was seen with Der p 1 and Der p 2: 155 (25.2%) and 161 (26.1%) respectively. This was followed by Der p 23; 117 (19.0%) and Der p 10; 33 (5.4%). The strongest relationship with both asthma and MSA was seen with sIgE to Der p 2 (Table 4). With higher levels of sIgE i.e., ≥3.5 the relationship between sIgE to Der p 1 and asthma became less significant. Analysing all the results for the sera where sIgE ≥3.5 IU/mL, sIgE to the components Der p 2 and Der p 23 had a significant relationship to both asthma and MSA while the comparable results for sIgE to Der p 1 were not significant (p = 0.30 and p = 0.20) (for significant relationships see bold values in Table 4).

**Table 3:** Relationship of sIgE Levels to cat allergen components among participants with current asthma “moderate to severe asthma” (MSA) and no asthma.

Cat components	No asthma (n = 516)	Asthma (n = 86)	Adjusted OR^c^ [+ or − C.I.]	p value^a^	MSA (n = 45)	Adjusted OR^c^ [+ or − C.I.]	p value^b^
>0.35 IU/mL							
Fel d 1	110 (21.3%)	43 (50%)	4.3 (2.5, 7.4)	<0.001	28 (62.72%)	7.1 (3.4, 14.7)	<0.001
Fel d 2	8 (1.6%)	9 (10.5%)	8.3 (2.6, 26.6)	<0.001	6 (13.3%)	11.0 (3.0, 40.1)	<0.001
Fel d 4	30 (5.8%)	22 (25.6%)	6.7 (3.2, 14.3)	<0.001	14 (31.1%)	10.5 (4.1, 26.5)	<0.001
Fel d 7	20 (3.9%)	17 (19.8%)	6.9 (3.1, 15.7)	<0.001	10 (22.2%)	9.0 (3.3, 24.2)	<0.001
>3.5 IU/mL							
Fel d 1	60 (11.6%)	31 (36%)	**4.6 (2.5, 8.4)**	**<0.001**	19 (42.2)	**6.2 (2.9, 13.2)**	**<0.001**
Fel d 2	4 (0.8%)	5 (5.8%)	9.5 (1.9, 47.8)	0.004	4 (8.9%)	21.8 (4.0, 117)	0.002
Fel d 4	7 (1.4%)	14 (16.3%)	**17.5 (4.9, 62.8)**	**<0.001**	10 (22.2%)	**29.4 (7.1, 121)**	**<0.001**
Fel d 7	11 (2.1%)	8 (9.3%)	6.3 (2.1, 19.1)	0.003	5 (11.1%)	7.9 (2.1, 29.4)	0.006
>50 IU/mL							
Fel d 1	11 (2.1%)	9 (10.5%)	**6.0** (**2.1, 17.2)**	**<0.001**	7 (15.6%)	**8.2 (2.6, 25.9)**	**<0.001**
Fel d 2	2 (0.4%)	1 (1.2%)	2.3 (0.1, 42.8)	0.4	1 (2.2%)	8.7 (0.4, 185)	0.2
Fel d 4	1 (0.2%)	3 (3.5%)	**11.3** (**0.6, 208.8)**	**0.01**	3 (6.7%)	**28.7 (1.6, 528)**	**0.002**
Fel d 7	0 (0.0%)	1 (1.2%)	–	–	1 (2.2%)	–	–

Bold values show adjusted odds ratios, confidence intervals, and p values (Chi^2^ test) for positive relationships to asthma and MSA identified in the text.

a Analysis is between no asthma vs asthma groups.

b Analysis is between MSA vs no asthma groups.

c Adjusted for child age at the Early Teen Visit, sex, race and ethnicity, BMI, delivery by cesarean section, household income, smoker in the home, cat at home, dog at home.

**Table 4:** Relationship of sIgE levels to mite allergen components among participants with current asthma “moderate to severe asthma” (MSA) and no asthma.

Mite components	No asthma (n = 530)	Asthma (n = 86)	Adjusted OR^c^ [+ or − C.I.]	p value^a^	MSA (n = 45)	Adjusted OR^c^ [+ or − C.I.]	p value^b^
>0.35 IU/mL							
Der p 1	123 (23.2%)	32 (37.25)	2.2 (1.3, 3.8)	0.006	19 (42.2%)	2.6 (1.3, 5.2)	0.007
Der p 2	124 (23.4%)	37 (43.0%)	3.0 (1.7, 5.1)	<0.001	24 (53.3%)	4.8 (2.4, 9.5)	<0.001
Der p 10	24 (4.5%)	9 (10.5%)	2.2 (0.8, 5.5)	0.03	8 (17.8%)	4.8 (1.8, 12.7)	0.002
Der p 23	91 (17.2%)	26 (30.2%)	2.5 (1.4, 4.4)	0.05	17 (37.8%)	3.2 (1.6, 6.7)	0.002
>3.5 IU/mL							
Der p 1	91 (17.2%)	19 (22.1%)	1.5 (0.8, 2.8)	0.3 NS	11 (24.4%)	1.7 (0.8, 3.8)	0.2 NS
Der p 2	100 (18.9%)	28 (32.6%)	**2.6 (1.5, 4.5)**	**0.004**	19 (42.2%)	**4.1 (2.1, 8.3)**	**<0.001**
Der p 10	10 (1.9%)	3 (3.5%)	1.5 (0.3, 7.2)	0.4 NS	3 (6.7%)	2.9 (0.6, 14.4)	0.07 NS
Der p 23	53 (10%)	17 (19.8%)	**2.4 (1.2, 4.7)**	**0.01**	11 (24.4%)	**3.0 (1.3, 6.8)**	**0.01**
>50 IU/mL							
Der p 1	11 (2.1%)	6 (7.0%)	5.5 (1.6, 18.2)	0.02	3 (6.7%)	6.0 (1.4, 25.9)	0.09
Der p 2	19 (3.6%)	10 (11.6%)	**4.5 (1.8, 11.3)**	**0.002**	6 (13.3%)	**5.6 (1.9, 16.2)**	**0.01**
Der p 10	2 (0.4%)	1 (1.2%)	–	–	1 (2.2%)	–	–
Der p 23	1 (0.2%)	2 (2.3%)	–	–	2 (4.4%)	–	–

Bold values show adjusted odds ratios, confidence intervals, and p values (Chi^2^ test) for positive relationships to asthma and MSA identified in the text.

a Analysis is between no asthma vs asthma groups.

b Analysis is between MSA vs no asthma groups.

c Adjusted for child age at the Early Teen Visit, sex, race and ethnicity, BMI, delivery by cesarean section, household income, smoker in the home, cat at home, dog at home.

### Specific IgG4 and sIgE to cat dander, cat exposure and the risk of asthma

**Fig. 3:**
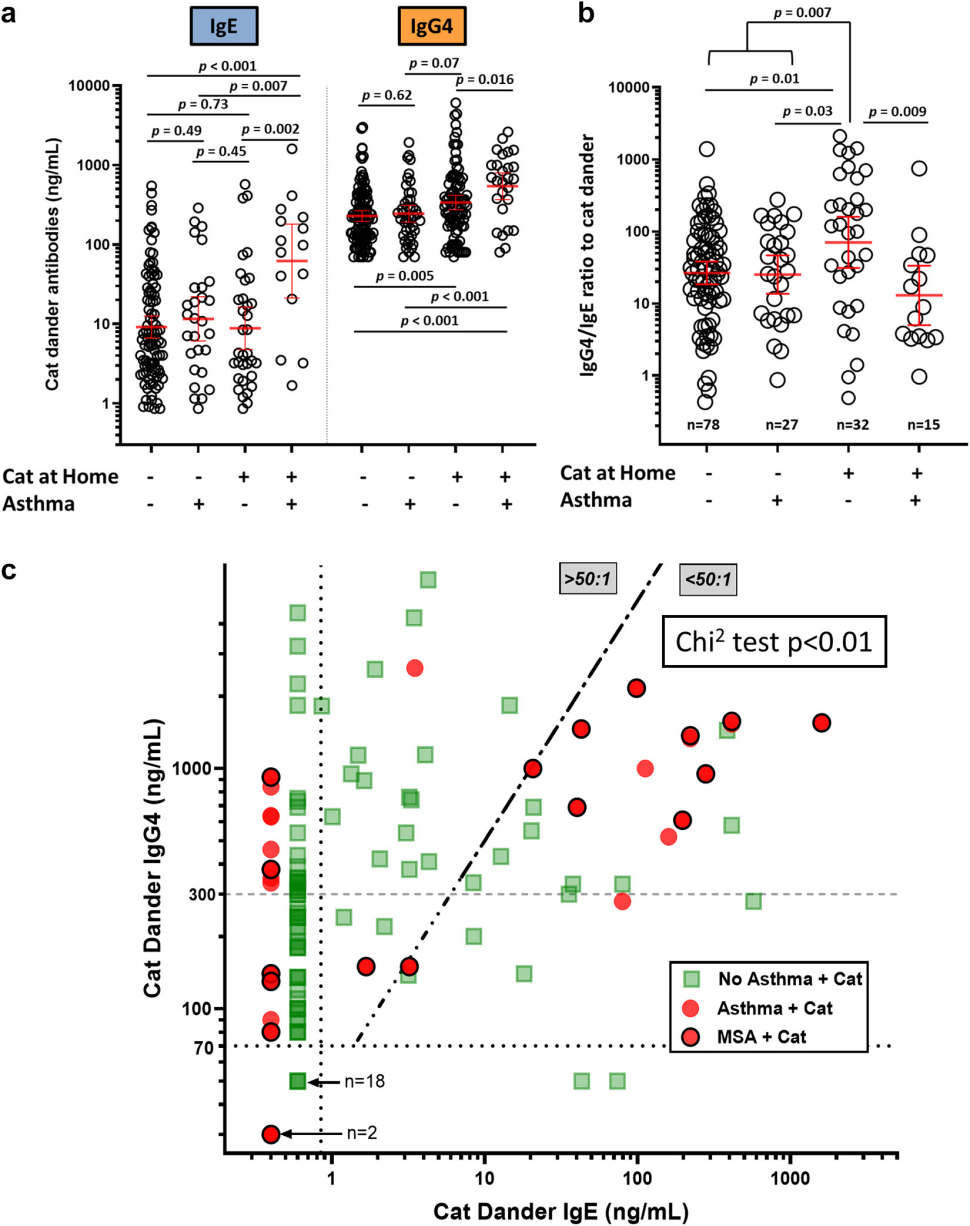
Antibodies to cat dander related to asthma and cat ownership. (a) Levels of slgE and sIgG4 expressed in ng/mL showing significantly higher levels of IgE in participants with asthma and a cat at home (p < 0.002, Mann–Whitney test). (b) Ratios of sIgG4:sIgE among those with positive values for both isotypes. The ratios were significantly lower among those with both asthma and a cat. (c) Plot of sIgG4 and slgE to cat dander, among 144 participants living in a house with a cat, showing participants with asthma (red dots); the subgroup with MSA (red dots with a black circle) and those without current asthma (green squares). The prevalence of asthma among those with a ratio <50:1, 13 of 27 (48%) was significantly higher than for those with a ratio >50:1, 2 of 20 (10%) (p < 0.01, Chi^2^ test). For Fig. 3c, we have included the current generic level of quantitation for sIgG4 of 300 ng/mL (horizontal dashed grey line) as used in some previous studies, as well as the 70 ng/mL (horizontal dotted line) used for our analyses of the data in the present study.

In the present study, assays for sIgG4 to cat extract were carried out on 348 sera including 85 participants with asthma. The highest levels of sIgG4 to cat allergens were observed for participants who had a cat at home and were also positive for sIgE to cat allergens (Fig. 3a). In order to further analyse the relationship of cat ownership to asthma, the results for sIgG4 and sIgE to cat allergens in sera from cat owners were expressed in ng/mL and presented both as ratios, and as an X, Y plot (Fig. 3b and c). For those with positive results for both isotypes and a cat in the home, the ratios of sIgG4:sIgE ranged from >500:1 to 1:1. Among those participants without asthma, the geometric mean of the ratio of sIgG4:sIgE for cat dander was 70.4:1, while the comparable ratio for the 15 participants with asthma was 12.9:1 (p = 0.009; Mann Whitney U test). Among those living in a house with a cat who had positive assay results for both isotypes and a ratio <50:1, 13 of 27 (48%) had asthma, by contrast among those with a ratio >50:1, two of twenty (10%) had asthma (p < 0.01; Chi^2^ test). Furthermore, of the 13 with asthma and a sIgG4:sIgE ratio <50:1 ten had MSA (Fig. 3c).

#### Specific IgG4 and sIgE to components of cat and mite allergens

##### Cat allergens

**Fig. 4:**
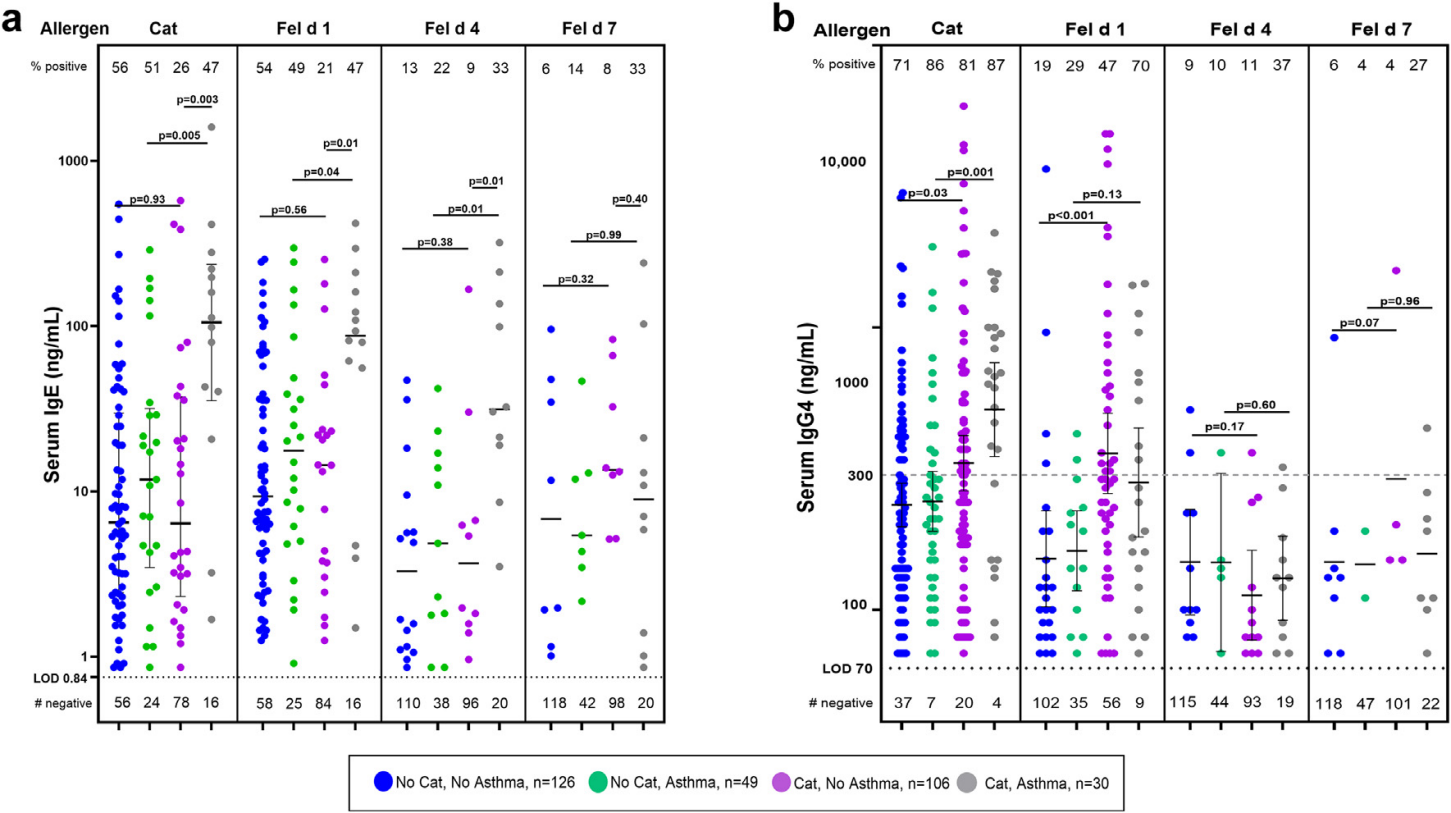
Specific IgE (a) and sIgG4 (b) levels to cat dander and three cat components stratified according to reported prevalence of a cat in the home and with or without asthma. Positive values are shown with GM values and 95% CI, and compared by Mann Whitney U test. For each set of measurements there are four groups; those with no cat and without current asthma (blue); those with no cat and asthma (green); those with a cat at home and no asthma (purple); and those with a cat in the home and asthma (grey). The reported presence of a cat in the home had a significant effect on the levels of sIgE to cat, Fel d 1 and Fel d 4. By contrast, the presence of a cat only had a significant effect on the level of sIgG4 to cat and Fel d 1. The prevalence of positive results for sIgG4 to Fel d 1, 52%, was significantly higher than that for Fel d 4 or Fel d 7 17% and 9% respectively (p < 0.01 and p < 0.001, Chi^2^ test). In addition, the presence of a cat in the home had no effect on levels of sIgG4 to Fel d 4 either among those with asthma (p = 0.6) or those without asthma (p = 0.17). In Fig. 4b, we have included the current generic level of quantitation of 300 ng/mL (horizontal dashed grey line) as well as the 70 ng/mL (horizontal dotted line) used for our analyses of the data in the present study.

**Fig. 5:**
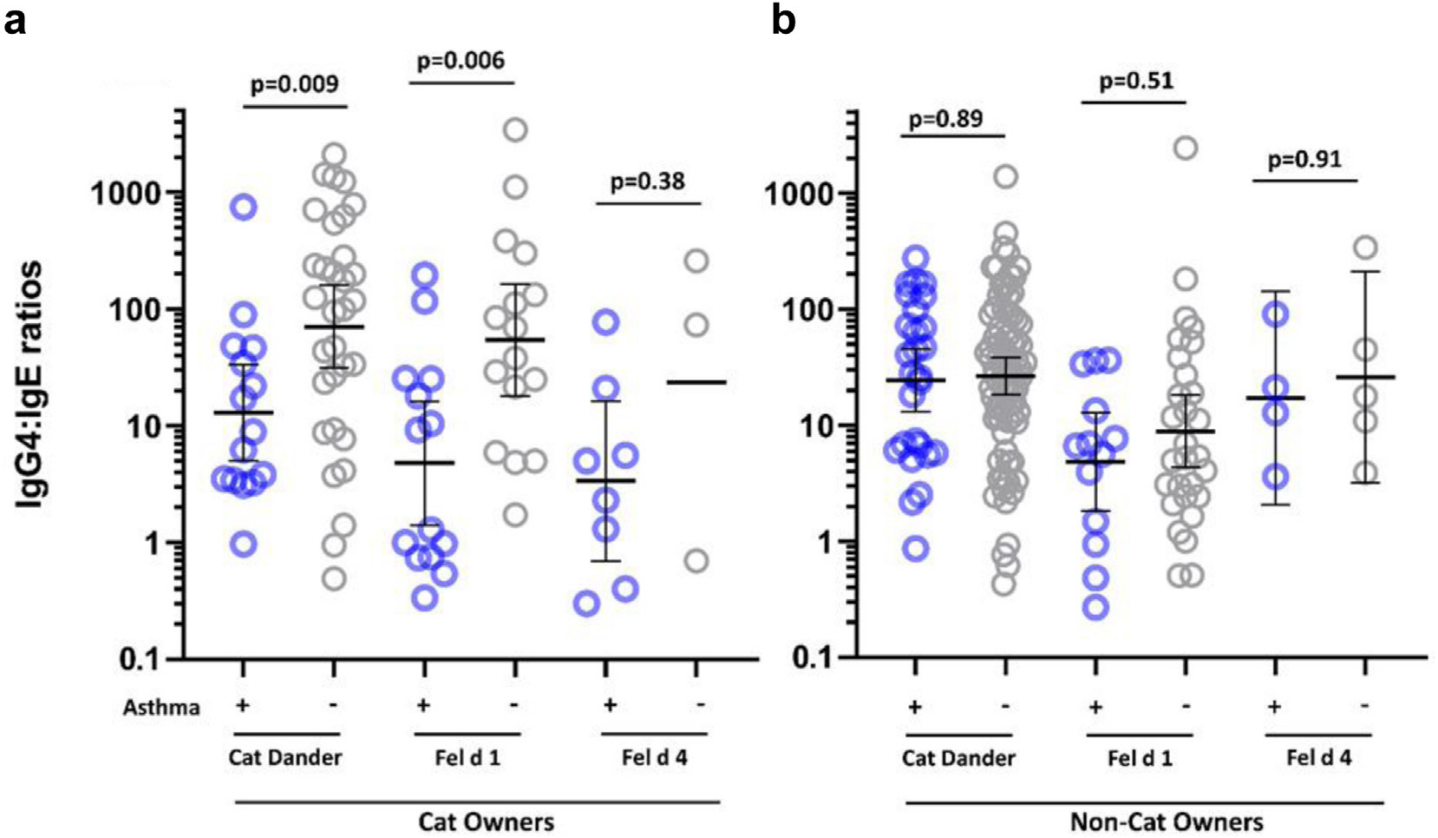
Ratios of specific IgG4: sIgE (both in ng/mL) presented as values of IgG4: IgE specific for three allergens, cat dander extract; Fel d 1, and Fel d 4. Among cat owners, (a) the ratios for cat dander and Fel d 1 are significantly lower for those with asthma; (p = 0.009 and p = 0.006 respectively, Mann–Whitney U test). Among those without a cat in the home, (b) the ratios are not significantly lower among those with asthma. The values for Fel d 4 reflect the fact that cat owners make very little IgG4 specific for this allergen either in the presence or the absence of a cat in the home.

Results for sIgG4 as well as sIgE for cat dander and the cat components were available on 310 participants, including 79 with asthma (Fig. 4a and b). In the results, the sIgG4 levels for cat dander were significantly higher among cat owners with or without asthma. Comparing the component results for both isotypes to the results for cat dander, it was clear that antibodies to Fel d 1 explained a large part of the IgG4 response to cat allergens. The IgG4 results for Fel d 4 and Fel d 7, showed a higher prevalence among cat owners, but there was no increase in GM of the levels of sIgG4 to these components among cat owners (Fig. 4b). The number of positive results for sIgG4 to Fel d 1 among the 135 cat owners tested was 70 (52%), compared to 23 (17%) for Fel d 4 (p < 0.001, Chi^2^ test). The ratios of sIgG4: sIgE for Fel d 1 were comparable to the same ratios for cat dander (Fig. 5). In keeping with the lower sIgG4 levels the ratios of sIgG4:sIgE for Fel d 4 among cat owners, who were positive for both isotypes, were not significantly different from the values for non-cat owners (p = 0.18) (see footnotes to Fig. 4, Fig. 5). The data for Fel d 2 are not included on Fig. 4 or Fig. 5 because the prevalence of sIgE was low, was not influenced by cat ownership, and there is no available data on exposure to this allergen.

The term cat ownership in Figs. 3 and 4 refers to reported cat ownership at the early teen visit. However, for 311 participants we also had data about cats at the mid-childhood visit, average age of 7. Of the 200 who had reported no cat at age 7, 35 reported having a cat in the house at the mid teen visit. For that group the sIgG4 was significantly higher than for those who reported no cat at both visits (p < 0.001, Mann Whitney U test) and was not significantly different from those who reported a cat at both visits (p = 0.26) (Fig S1). Thus, the high values for sIgG4 to cat allergens at the mid teen visit appear to reflect the effects of cat exposure over the previous 5 years.

##### Mite allergens

**Fig. 6:**
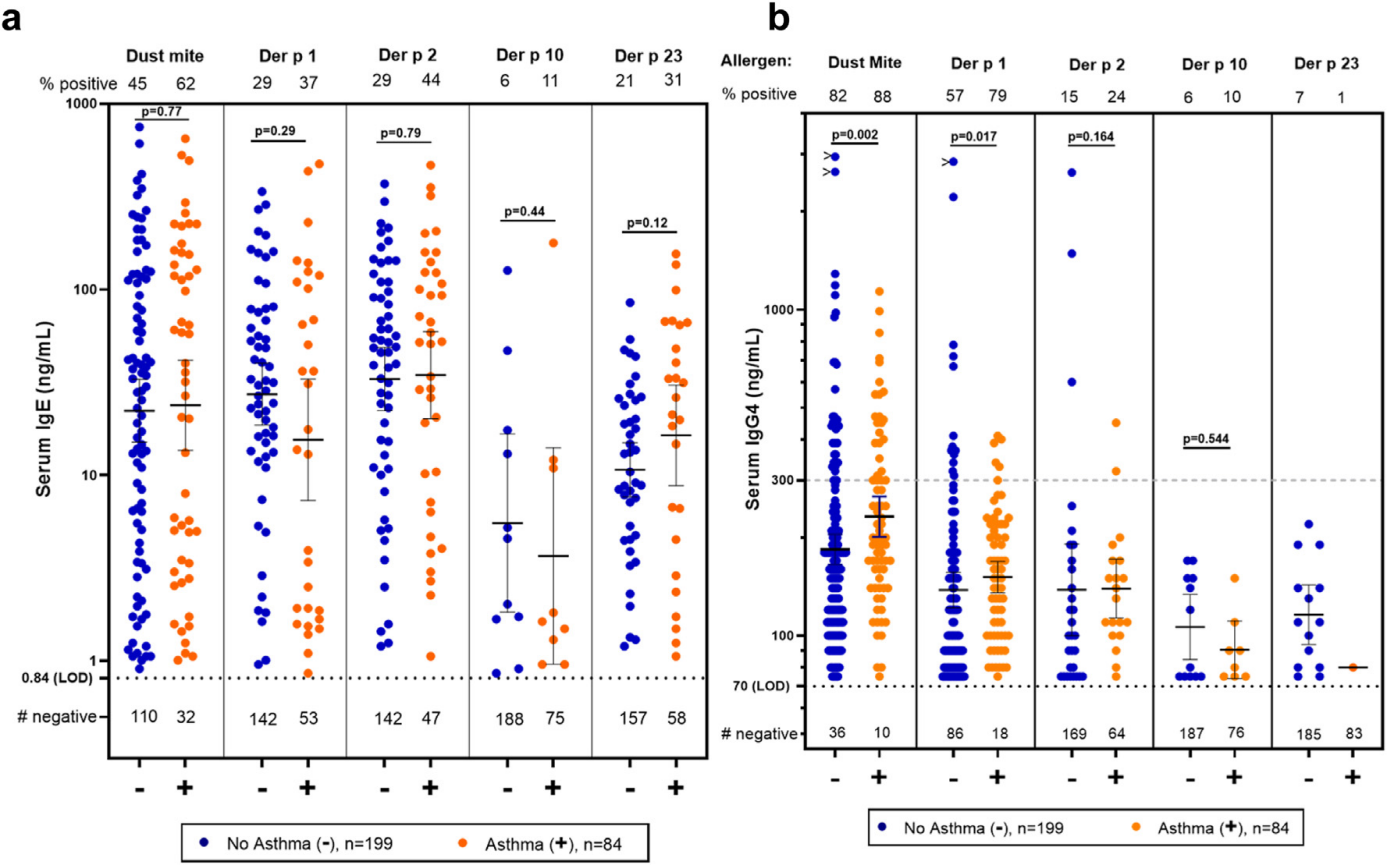
Values for sIgE and sIgG4 antibodies (both in ng/mL) to dust mite and the mite allergen components Der p 1, Der p 2, Der p 10, and Der p 23 were available for 283 participants. (a) Levels of sIgE for 199 participants without current asthma and 84 with current asthma. (b) Levels of IgG4 in ng/mL are shown for the same 199 participants without asthma and 84 with current asthma. In Fig. 6b, we have included the current generic level of quantitation of 300 ng/mL (horizontal dashed grey line) as used in some previous studies, as well as the 70 ng/mL (horizontal dotted line) used for our analyses of the data in the present study.

The values for both sIgE and sIgG4 were available for 84 participants with asthma and 199 participants without asthma (Fig. 6a and b). The overall prevalence of sIgG4 to mite extract was 85%, and the occurrence of sIgG4 to Der p 1 was also high with 179 of 283 positive (63%). The corresponding values for Der p 2 and Der p 23 were 18% and 5% respectively (Table 5). In keeping with published data, the prevalence of positive IgG4 antibodies was higher among those who had sIgE to the same allergens. For mite, Der p 1 and Der p 2, this was true among IgE positive individuals who had asthma, i.e., 94%, 90%, and 50%, respectively. However, there were two clear effects that have not previously been reported. For Der p 2, the prevalence of sIgG4 was significantly lower among those without sIgE to Der p 2: 6/189 (3%) compared to those with sIgE to Der p 2, 44/94 (47%) (p < 0.001, Chi^2^ test) (Table 5). For Der p 23, all groups had very low prevalence of sIgG4, and only one of the 26 participants with asthma who were sensitised to Der p 23 also had detectable sIgG4. Given the progressive fall in the prevalence of sIgG4, and the relative consistency of sIgE to mite, Der p 1, Der p 2, and Der p 23, it was not surprising that the number of sera with both sIgE and sIgG4 positive for each component declined (Table 5). The sIgG4 results for Der p 10 were comparable to those for Der p 23 and not significantly associated with asthma (Fig. 6b). The scale of the change in positive values for sIgG4 among participants with asthma was remarkable; for mite extract, Der p 1, Der p 2, and Der p 23 the ratios were 49/52; 28/31; 19/37; and 1/26 respectively (Table 5). Clearly those results raise questions about what feature or features of the allergens are relevant to the production of sIgG4. Our current hypothesis is that quantitative exposure to, or abundance of, these allergens could be relevant to progressively lower prevalence of detectable sIgG4 to Der p 1, Der p 2, and Der p 23 (Table 6). Perhaps the most important feature of those results is that, for the mite components with the stronger association with asthma i.e., Der p 2 and Der p 23 (see bold values in Table 4) the prevalence of sIgG4 to Der p 2 and Der p 23 was 18% and 5% respectively.

**Table 5:** Prevalence of specific IgG4 to dust mite and mite components in relationship to asthma among subjects with or without specific IgE to mite extract or the relevant mite component.

	Total group n = 283	Specific IgE to dust mite or dust mite components		Comparison of IgE + and IgE − groups^b^
		Positive	Negative	
	sIgG4 positive	sIgG4 pos/sIgE pos	sIgG4 pos/sIgE neg	
Dust mite				
Overall	**237/283***(84%)*	**126/141***(89%)*	**111/142***(78%)*	0.011
Asthma	**74/84***(88%)*	**49/52***(94%)*	**25/32***(78%)*	0.027
Non-asthma	**163/199***(82%)*	**77/89***(87%)*	**86/110***(78%)*	0.13
Der p 1				
Overall	**179/283** (*63*%)	**67/88***(76%)*	**112/195***(57%)*	0.003
Asthma	**66/84***(79%)*	**28/31***(90%)*	**38/53***(72%)*	0.045
Non-asthma	**113/199***(57%)*	**39/57***(68%)*	**74/142** (52%)	0.036
		ap = 0.51		
Der p 2				
Overall	**50/283***(18%)*	**44/94***(47%)*	**6/189***(3%)*	<0.001
Asthma	**20/84***(24%)*	**19/37***(51%)*	**1/47***(2%)*	<0.001
Non-asthma	**30/199***(15%)*	**25/57***(44%)*	**5/142***(4%)*	<0.001
		ap < 0.001		
Der p 23				
Overall	**15/283***(5%)*	**6/68***(9%)*	**9/215***(4%)*	0.14
Asthma	**1/84***(1%)*	**1/26***(4%)*	**0/58***(0%)*	0.13
Non-asthma	**14/199***(7%)*	**5/42***(12%)*	**9/157***(6%)*	0.16
		ap < 0.001		

a p values for the comparison of IgG4/sIgE for each component among participants with asthma compared to the same value for sIgG/sIgE to dust mite extract among those with asthma.

b Statistics for sIgG4/sIgE for mite and for each of the components comparing participants with positive values for IgE to participants without IgE for each component. Statistics were analysed using Chi^2^ test.

**Table 6:** Estimates of exposure to allergens from cat or dust mite: airborne and floor dust.

	Component	Airborne	Floor dust/gram of dust	Refs
Cat	Fel d 1	Up to 2 μg/m^3^	Up to 1000 μg/g	^22^
	Fel d 4	No measurements	∼4 μg/g	^24^^,^^33^
Mite	Der p 1	Up to 70 ng/m^3^	0.03–50 μg/g	^22^
	Der p 2	Up to 30 ng/m^3^	0.2–5 μg/g	^22^
	Der p 23	Too low to measure	≤1 μg/g	^30^

## Discussion

The possibility that children raised in a house with a cat were less likely to become allergic to cats was first proposed in 1999. Similar observations were made in several studies spanning across many countries. Our own data has come from cross sectional studies in the USA and New Zealand as well as from a prospective study in Northern Sweden.^7^^,^^10^^,^^37^ The data presented here provides further detail of the form of tolerance that occurs in many young people who are living in a home with a cat.^6^^,^^7^^,^^37^ The current evidence is that a ratio of sIgG4:sIgE to cat dander ≥50:1 is common in the sera of the participants in this study and is negatively associated with the risk of asthma. In addition, we confirm that this effect of cat ownership relates specifically to the major cat allergen Fel d 1. Two further significant additions to understanding the relationship between exposure to an inhalant allergen and both the immune response and asthma have come from the present studies. Firstly, that there was no apparent effect of cat ownership on the levels of IgG4 responses to either of the two lipocalin allergens Fel d 4 and Fel d 7 or the albumin Fel d 2. Secondly, that a similar phenomenon occurs in relation to mite allergens in that a high prevalence of sIgG4 is seen with Der p 1 (67%), while antibodies of this isotype are less common with Der p 2 (20%) and rare with Der p 23 (5%). Most studies on the relevance of mite allergen exposure in childhood, including two in the USA, have reported a positive association between high exposure and a higher prevalence of both sIgE and asthma.^4^^,^^35^ However, there have been a few studies from countries known to have high exposure to mite that have reported lower prevalence of sensitisation to mite allergens among children with the highest levels of allergen exposure.^38^ In particular, the study on young children in Sydney reported significantly decreased sensitisation and asthma among children living in houses with the highest quintile of exposure i.e., ≥23 μg Der p 1/gram of dust.^38^ In the Viva cohort, mite allergen was measured in houses at age 7 years, but the highest levels of Der p 1 were lower than the levels reported from Australia. What may be relevant to our present results is that Der p 1, which is known to be the most abundant mite allergen in dust from houses and airborne, was associated with a high prevalence of sIgE, but had a much higher prevalence of sIgG4 and a lower association with asthma than that seen with sIgE to Der p 2 or Der p 23 (Tables 4 and 5).

Interestingly, the higher prevalence and levels of sIgG4 antibodies were seen with the first allergens purified i.e., Fel d 1 (1974) and Der p 1 (1979).^20^^,^^21^ In each case, the original purification was carried out using gel filtration and electrophoresis or ion exchange. Those physicochemical processes favoured purifying an allergen that was present at higher concentration in the extract. Indeed, the available data supports the view that Fel d 1 and Der p 1 represent the highest exposure from these two sources (Table 6).^35^ Although the levels of sIgG4 to cat extract and Fel d 1 may be high compared to other inhaled allergens, they are modest by comparison with the quantities of sIgG4 found to proteins from cow's milk or wheat in patients with eosinophilic esophagitis (EoE) (Fig S2). In those cases, quantities of sIgG4 ≥10 μg/mL were not unusual and we used that level as a cutoff for evaluating the risk of EoE.^27^ Needless to say the quantities of milk or wheat proteins eaten are measured in grams/day. Thus, there is good evidence from several types of studies that production of high levels of sIgG4 in this age group is dependent on high exposure.

It is worth emphasising that primates have been evolutionarily separate from dust mites for as long as 600 million years, while we have only been separate from other mammals for 60 million years. Thus, it should not be a surprise that some mammalian proteins are not well recognised by the human immune system. This “problem” was pointed out by Dr. Spitzauer in Vienna who described mammalian proteins and especially albumins such as Fel d 2 as “on the borderline between foreign and self”.^39^ The dominance of sIgE against Fel d 1 and Der p 1 may primarily be a consequence of their abundance in the respective sources. However, each of these proteins has its own characteristics: Fel d 1 is present in all cat species and has only limited homology with other mammalian proteins; by contrast Der p 1 is a potent cysteine protease and many authors have argued that the enzymatic activity of this protein plays an important role in its ability to initiate the IgE response to dust mite allergens.^40^, ^41^, ^42^ Our data supports the view that the “minor” components of both dust mite and cat extracts can have a greater role in asthma because they induce smaller quantities of sIgG4 antibodies. In turn, this suggests that component spreading for sIgE production can occur with levels of exposure to an allergen that are too low for effective production of sIgG4 to the same proteins. In relation to allergic disease, the term epitope spreading has been used to refer to two separate phenomena. The first is intra-molecular, which is correctly epitope-spreading, where there is a progressive increase in the number of epitopes recognised by sIgE on the surface of a given protein molecule. The second is inter-molecular spreading from a “major” allergen to other allergens from the same source, better called component spreading.^34^^,^^43^ However, only limited data has been reported on component spreading for allergen specific IgG4. Indeed, the only clear data relates to the allergens of peanut.^44^

We have chosen not to measure sIgE and sIgG4 to other allergens that have been purified from these two sources or other sources. There has been a major increase in the number of allergens identified in cat and mite extracts, which has been made possible by cloning of these proteins (see www.allergen.org).^45^ It is important to recognise that cloning of a protein gives no evidence about its abundance in the environment. In some cases, the evidence that these proteins are clinically important allergens is not very strong, and even where there is excellent evidence about the relevance of the proteins to sIgE production, in most cases there are no measurements of exposure either in allergen extracts or in houses. A recent paper reported mass spectroscopy data on proteins in house dust with striking evidence about the quantities of Der p 2. However, that data has not been applied to airborne allergens and does not provide evidence about the antigenicity of the protein sequences identified.^46^ There has been a description of the relevance of Der p 37 to asthma, but at present, there are no measurements of exposure to this novel allergen and it is not available on a high capacity solid phase.^47^

A limitation of our results is the lack of current exposure measurements in the homes of our participants. The cohort was initially enrolled in Eastern Massachusetts, participants are now more scattered across the region. Because of the COVID-19 pandemic, we would not have been able to do home visits in the last 3 years. However, in addition there are many difficulties in interpreting measurements made in a house. We consider that cat ownership is a good surrogate for high exposure to the cat allergen Fel d 1, but that there is much less evidence about the effect of cat ownership on airborne exposure to other cat allergens.^23^ For mite allergens assays of specific proteins in micrograms per gram of dust are the most easily standardised, but they are a poor surrogate for assays of airborne allergens.

There are several aspects of housing management or design that may have had important effects on the allergen exposure of children in the Viva cohort. The most obvious is the progressive expansion of home air conditioning (A/C) over large areas of the United States. In particular, the availability of central cooling makes it possible to close houses as soon as the temperature rises, often as early as May. This makes it possible to hide from pollen in the middle of summer and early fall. By contrast, in areas where A/C is not widespread such as Northern California grass pollen can become a major source of allergens in house dust. Equally in Virginia 40 years ago the use of window fans, which could dramatically increase pollen exposure, was not unusual, Certainly, the introduction of central A/C may be part of the explanation for the fact that sIgE to grass and ragweed pollen are less relevant to asthma in the Viva cohort than they were in the past. Equally it may explain why sensitisation to the pollens of oak and birch which pollinate in Feb–April are now of greater relevance. Another effect of A/C is to decrease overall humidity in a house which may help to reduce the growth of mites and fungi in houses.

In conclusion, using a quantitative assay for sIgE and sIgG4 to a range of inhalant allergens and also to component allergens of cat and dust mite, we have studied the relevance of these allergens to asthma among ∼600 participants of the Viva cohort in Boston. For both cat and dust mite, the components included the first recognised major allergens i.e., Fel d 1 and Der p 1 and also a second or third allergen for which we had information about exposure. In keeping with our previous data, many of the participants living in a house with a cat had high levels of sIgG4 to cat extract and Fel d 1.^7^^,^^13^^,^^15^ Although there were a large number of participants who had made both sIgE and sIgG4 but did not have asthma, the risk of asthma was high among those with a ratio of sIgG4 to sIgE ≤30:1 in ng/mL for cat dander. For the lipocalin allergen Fel d 4 quantitative exposure is less and cat ownership had no quantitative effect on sIgG4 to this allergen, but was associated with increased sIgE. Comparing the three best defined allergens derived from dust mite, Der p 1, Der p 2 and Der p 23 there was an inverse relationship between their relative abundance and the associated risk of asthma. In addition, the prevalence of sIgG4 to these three components of mite significantly decreased for those which are present in lower quantities in house dust. The implication is that abundant allergen components (such as Fel d 1 and Der p 1) representing the highest levels of exposure, can induce an antibody response with lower levels of sIgE and higher levels of sIgG4 which we might now call “a modified Th2 related antibody response”. That response can have a controlling effect on the relevance of those specific allergens to asthma.^7^^,^^12^ By contrast, for those allergens that are significantly lower in the indoor environment, such as Fel d 4, Der p 2, and Der p 23, their relationship to asthma may be high relative to their abundance because they induce less sIgG4.

## Contributors∗

• Dr. Behnam Keshavarz has extensively contributed to running the majority of the IgE and IgG4 assays, as well as analysing the data.
• Dr. Jeffrey Wilson has contributed extensively to the analysis of data and writing on the manuscript.
• Sheryl Rifas-Shiman is a statistician and epidemiologist and had full access to the data at all stages of this study.
• Samuel Ailsworth carried out a significant part of the IgG4 assays and developed methods and analysis of data.
• Dr. Joanne Sordillo has contributed important understanding of the Viva cohort including the criteria for diagnosis and previous data on asthma in the participants.
• Lisa Workman is the senior technician in the laboratory at UVA and was responsible for establishing the use of the sIgG4 assay in our lab. She has had full access to the data.
• Dr. Martin Chapman has collaborated with our group for many years in the evolution of studying allergen exposure and is responsible for much of the data in Table 6.
• Jonas Lindholm is a full-time employee of Phadia/Thermo Fisher who has provided extensive advice about the assays, in particular about IgG4 assays and analytic aspects of IgG4 measurements and cutoff levels (or LOD).
• Dr. Emily Oken is one of the founders of the Viva cohort working closely with Dr. Diane Gold. She has provided information about the cohort and been a great help in obtaining specimens.
• Dr. Diane Gold is the co-head of the Viva cohort and was responsible for the addition of analysis of allergic disease to the Viva cohort at age 3, and has been a close colleague in analysis and writing of the current manuscript.
• Dr. Thomas Platts-Mills has been the head of the allergic diseases program at the UVA for over 30 years. His research on dust mite allergens started in the UK in the 1970s. He wrote the 2001 paper in the Lancet on the effects of cat ownership. He designed the present studies with Dr. Diane Gold, Dr. Behnam Keshavarz, and Dr. Jeffrey Wilson, and is responsible for the current revision.

∗All authors have read and approved the final version of the manuscript.

## Data sharing statement

Project Viva data are not all publicly available because historic consents have not covered public data sharing. The deidentified data in this article may be accessible upon reasonable request, subject to approval from both the Project Viva team and the Institutional Review Board. To enquire, please contact VivaROADMaP@hphci.harvard.edu. A formal protocol outlining the procedures for individual investigators seeking to use Project Viva data is available at https://www.projectviva.org.

## Code sharing

The code used in this article is available on reasonable request. To request access, please contact VivaROADMaP@hphci.harvard.edu.

## Declaration of interests

The primary funding source for the experimental studies is the National Institute of Allergy and Infectious Diseases of the NIH and the specific grant is A1-20565-37 on which Dr. Platts-Mills is the principle investigator. Dr. Platts-Mills received funding to attend meetings from Phadia and Thermo Fisher, and was on the data safety monitoring board NIAID (2014–2023). In addition, we have had extensive support in the form of assay materials for IgE and IgG4 antibodies from Phadia/ThermoFisher, Upsala, Sweden. That support includes no restriction on the studies carried out and has no restrictions on publication. The main funding for the Project Viva cohort comes from NIH grants R01HD034568 and R24ES to Emily Oaken and Diane Gold. None of the authors have significant personal conflicts in relation to the findings of this article. However, Martin Chapman, PhD is owner of the company InBio and has stocks in InBio, he also receives a grant NIH NIAID R01AI077653-13. Jonas Lidholm, PhD is employed full-time by Phadia/ThermoFisher and has stocks in Thermo Fisher Scientific. Jeffrey Wilson receives material support from Thermo-Fisher Scientific for research assays unrelated to current work.
