# Supplementary material for: High risk of asthma among early teens is associated with quantitative differences in mite and cat allergen specific IgE and IgG4: a modified Th2 related antibody response revisited
Source: eBioMedicine. 2025 Feb 1;112:105556. doi: 10.1016/j.ebiom.2024.105556 (PMC11840499; doi:10.1016/j.ebiom.2024.105556)
Supplement: Figures S1 and S2 [file mmc1.docx]

**Supplementary Figures**


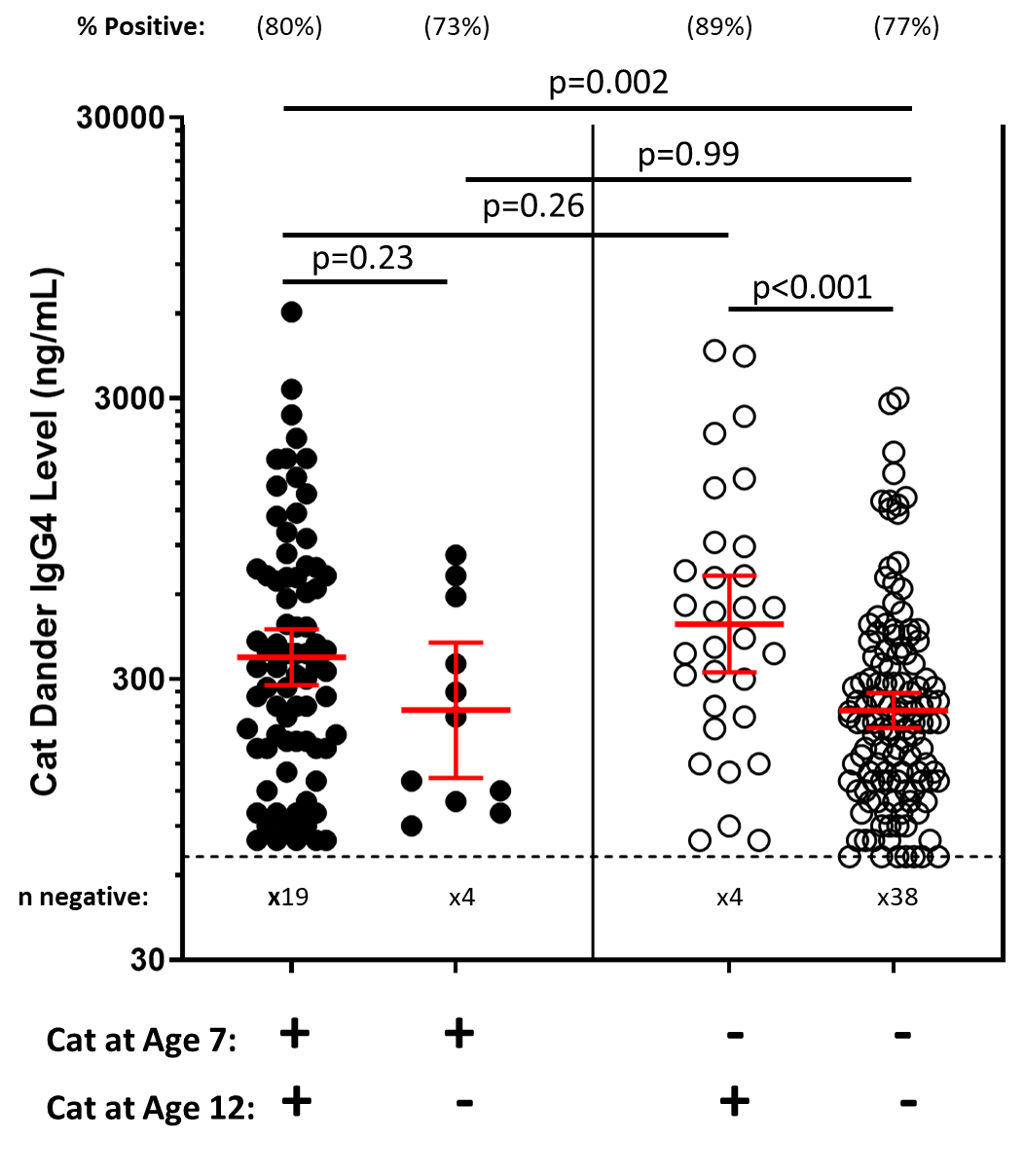


Figure S1: The prevalence and levels of sIgG4 to cat extract at age 12, stratified by reported cat ownership at age 7 and/or age 12. Those with no cat at age 7 and no cat at age 12 (n=165) had significantly lower sIgG4 than those who reported a cat at both visits (p=0.002). Those sera from individuals who had no cat at age 7 and reported the presence of a cat at age 12 years (n=35) had significantly higher sIgG4 to cat than those who continued to live in a home without a cat at age 12 (p<0.001). Furthermore, these values for sIgG4 were not significantly different from the levels in individuals who reported the presence of a cat at both visits (n=96) (p=0.26). The number of cases where a cat was present at age 7 but not at age 12 was much lower than the other groups (n=15). Although a minor decrease in sIgG4 was seen in this group, it was not significant (p=0.23)


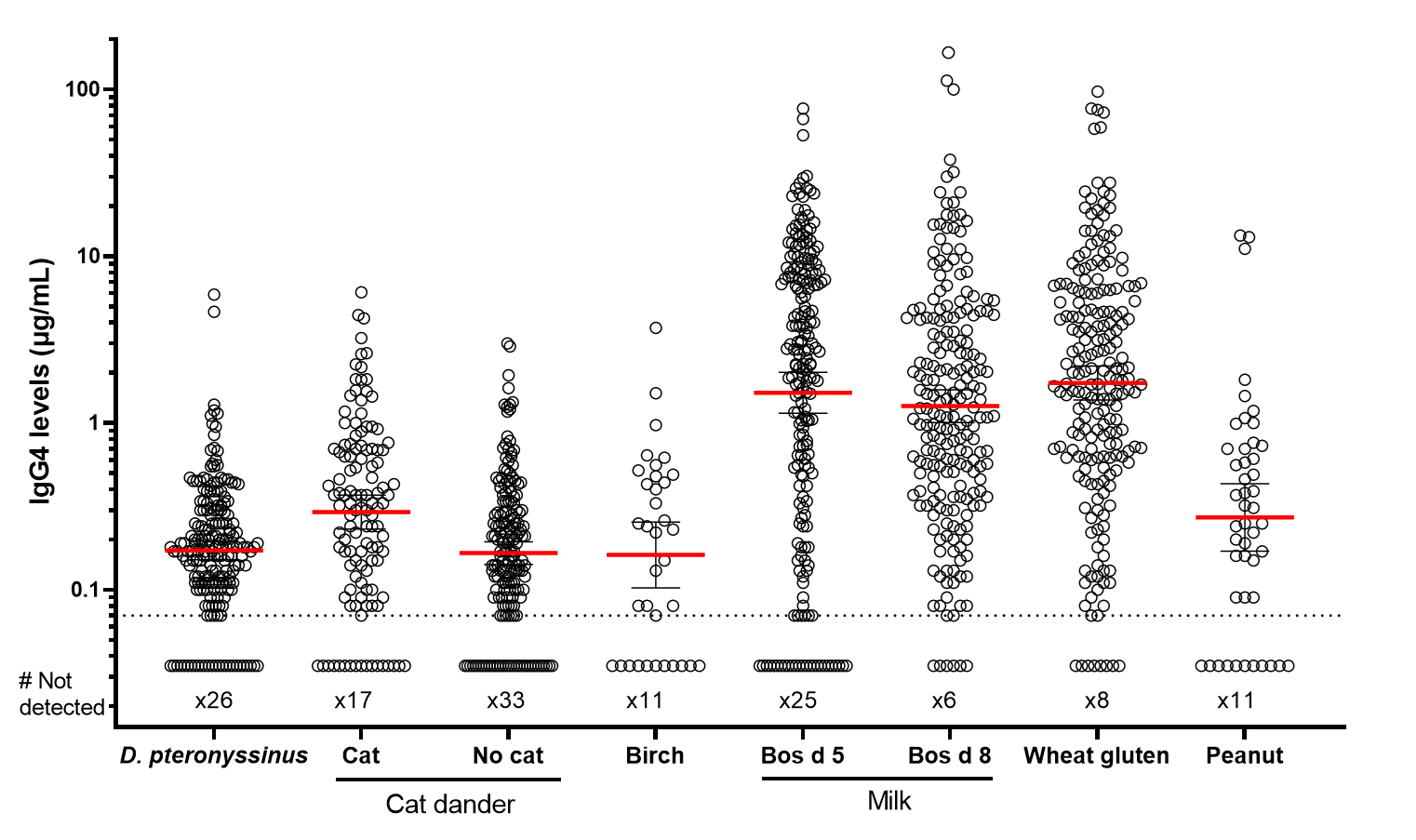


**Figure S2.** Geometric mean of positive levels of sIgG4 to select inhalant and food allergens (Bos d 5: β-lactoglobulin, Bos d 8: casein)

Values for Bos.d.5, Bos.d.8, wheat, gluten, and peanut are from assays on 200 random controls from Schuyler et al, JACI 2018 (Ref 27).

Values for mite, cat, no cat, and Birch are from assays on the present cohort. The values on Birch are from 35 random subjects from the present cohort. For dust mite and birch the prevalence of values > 1µg/mL was very low.
